# Clarifying solvent effect during photocatalytic glycerol conversion on TiO_2_/GQD as selective photocatalyst

**DOI:** 10.1038/s41598-023-48781-3

**Published:** 2023-12-09

**Authors:** Sara Hassan, Dalia R. Abd El-Hafiz, E. S. Abdullah, Mostafa M. H. Khalil

**Affiliations:** 1 Egyptian Petroleum Research Institute EPRI, Nasr City, Cairo Egypt; 2 Chemistry Department, Faculty of Science, Ain Shams University, Abbassia, Cairo 11566 Egypt

**Keywords:** Catalysis, Photochemistry, Chemical engineering

## Abstract

Nowadays, dealing with the growing chemical and energy demands is important without compromising the environment. So, this work studies photocatalytic glycerol conversion (as biomass derivativ feedstock) into value-added products using an eco-friendly synthesized catalyst. Graphene quantum dots (GQDs) were prepared from available/cheap precursors like glucose via the hydrothermal method and used as a support for TiO_2_. TiO_2_/GQDs were characterized via different analytical techniques, revealing very small particle sizes of ~ 3–6 nm with a large surface area of ~ 253 m^2^/g and a band gap of ~ 2.6 eV. The prepared photocatalyst shows good efficiency during photocatalytic glycerol conversion to dihydroxyacetone (DHA). Different reaction conditions were tested: reaction time, catalyst amount, presence of oxidant (H_2_O_2_), and biphasic media (aqueous/organic phases). Comparing a monophasic (H_2_O) photoreactor with a biphasic reactor containing 90% organic phase (ethyl acetate) and 10% aqueous phase (H_2_O and/or H_2_O_2_) indicates that the presence of H_2_O_2_ increases glycerol conversion and liquid selectivity to reach 57% and 91%, respectively after 120 min. However, it still suffers a low DHA/GA ratio (2.7). On the other hand, using a biphasic reactor in the presence of an H_2_O_2_ oxidant increases the DHA/GA ratio to ~ 6.6, which was not reached in previous research. The formation of H_2_O/H_2_O_2_ as micro-reactors dispersed in the ethyl acetate phase increased the average light intensity effect of the glycerol/photocatalyst system in the micro-reactors. Unlike previous work, this work presents a facile way to prepare eco-friendly/cheap (noble metal free) photocatalysts for glycerol conversion to ultrapure DHA using a biphasic photoreactor.

## Introduction

Glycerol (1,2,3-propanotriol) is an organic compound found in most animal and vegetable oils and fats containing three hydroxyl groups, defining it as alcohol. Glycerol is primarily obtained as a byproduct during the transesterification process for biodiesel production. For every 10 kg of biodiesel produced, approximately 1 kg of crude glycerol is produced^1^. The amount of glycerol that can be used as an addition in the food and drug production industries is large since it is a harmless and environmentally beneficial product. Glycerol is a multifunctional compound that can be refined into thousands of fine chemicals such as 1,3-dihydroxyacetone (DHA), acrolein, ethanol, and propylene glycol^2,3^^.^

Glycerol conversion occurs via different processes, including photocatalytic reforming and others. Photocatalysis can also be used in environmental remediation applications, such as breaking down toxic organic compounds into mineralized products, destroying airborne pollutants, waste-water treatment and the breakdown of industrial effluents^4,5^.

A photocatalyst is any material with photocatalytic qualities, that is, the ability to encourage and accelerate specific chemical reactions when stimulated by light of a specified wavelength^6^ Due to its wide bandgap, photochemical stability, strong oxidizing abilities, superhydrophilicity, chemical stability, long durability, nontoxic nature, biocompatibility, photo corrosion-free nature, low cost, transparency to visible light, and titania (TiO_2_) is one of the most commonly used photocatalysts^7,8^. Nevertheless, TiO_2_'s intrinsic optical and electrical characteristics are insufficient for achieving appropriate catalytic efficiency for two reasons: its restricted ability to use solar energy and the quick recombination of photo-generated electron–hole pairs during catalytic processes^9–12^. So, various methods for increasing carrier production, and charge separation^13^. One is TiO_2_ coupling and doping with metals, nonmetals, ions, or other semiconductors, creating trap sites and aiding in charge carrier separation. The coupling of TiO_2_ nanoparticles with graphene quantum dots (GQDs) will be the focus of this paper^14^. The carbon-based nanoparticles known as graphene quantum dots (GQDs) have exceptional chemical, physical, and biological characteristics that make them successful in various applications. GQDs are small chunks or fragments of graphene with lateral dimensions less than 100 nm in one or more layers^15,16^. They are called zero-dimensional (0D) with a diameter of ~ 10 nm. The presence of a graphene lattice, which gives excellent crystallinity and carbon structure, is a crucial component of GQDs^17,18^ GQDs offer exceptional qualities such as ultraviolet-blue to green luminescence, great photostability, biocompatibility, chemical inertness, high surface area, nontoxic, being environmentally benign, and cost-effective synthesis methods because of their quantum confinement and edge effects^18,19^. GQDs can replace conventional QDs in various applications, including photocatalysis, sensors, light-emitting diodes (LEDs), and energy conversion or storage devices^20,21^. Based on the aforementioned, using GQDs to alter TiO_2_ nanoparticles can provide intriguing materials advantageous for photocatalysis aapplications^22,23^.

From another point of view, most of the photocatalysis process occurs in aqueous media^24^. Water is a common solvent in the photocatalysis process because it is a transparent, green, radical-forming solvent. Non-aqueous photocatalysis is not fully considered. Using non-aqueous solvents leads to different solvent/photocatalyst interfaces than those formed with water, resulting in different valence and conduction bands than those reported using water solvents, which affect both the activity and selectivity of photocatalysts. This can also be attributed to the change in how reactants/intermediates adsorb to photocatalyst surface, and then redox reactions will occur^25,26^^.^

Moreover, non-aqueous solvents can also affect the light distribution caused by including an active/inactive co-solvent^27^. Ethyl acetate, which is made from biomass, is one of the commonly used co-solvents. Ethyl acetate can eliminate contaminants from crude glycerol to prevent photocatalyst poisoning. Imbault and coworkers 28 used the first biphasic photoreactor to increase DHA selectivity using ethyl acetate as a co-solvent. It was observed that biphasic reactors are better than monophasic ones, increasing the photocatalytic efficiency and improving the DHA/GA (glycerol aldehyde) ratio to ~ 3. Nevertheless, the process still needs further study.

We present a model of biphase reactor responsive photocatalysts, including TiO_2_/GQDs nanocomposites, for studying the selective photocatalytic conversion of glycerol to dihydroxyacetone (DHA), which uses H_2_O_2_with (ethyl acetate/H_2_O), which has never been used before. The GQD powder was made hydrothermally from glucose powder using an economical and environmentally friendly method. GQDs have exceptional qualities, such as high chemical stability, electrical conductivity, good solubility in common solvents, and photoinduced electron-transfer properties.

## Methods

### Materials

Titanium isopropoxide (TTIP, 97.0%), ethanol (99.9%), ethylene glycol (EG, 99.0%), hydrochloric acid (37.0%), methanol, glucose powder, glycerol (> 99%) and hydrogen peroxide (H_2_O_2_) were purchased from Sigma-Aldrich Co. Cetyl trimethyl ammonium bromide (CTAB, 99.0%) was purchased from BDH.

### Preparation of graphene quantum dots (GQDs)

50 mL of deionized water dissolved 6 g of glucose powder. A one-step hydrothermal process was used to process the mixture in a 100 mL Teflon-lined stainless-steel autoclave at 200 °C for eight hours. To get GQDs powder.

### Preparation of TiO_2_ nanoparticles

Under vigorous stirring, 0.5 mol of TTIP were added to 10 mL of HCl solution. After infusing the solution with 50 mL of EG, the solution was supplemented with 0.003 mol of cetyl trimethyl ammonium bromide, dissolved in 5 mL of distilled water. For an hour, the mixture was agitated. Then, the solution was put into a 100-mL autoclave and cooked for 20 h at 150 °C. Then, after centrifuging the produced TiO_2_ NPs, the residue was repeatedly cleaned with distilled water and 100% ethanol before being dried for two hours at 100 °C and annealed for three hours at 400 °C.

### Preparation of TiO_2_/GQDs nanocomposites

TiO_2_/GQD nanocomposites were created using hydrothermal techniques. This procedure combined 0.25 g TiO_2_ NPs and 0.05 g GQDs with 20 mL distilled water and 10 mL absolute ethanol. The mixture was agitated at room temperature for 30 min to create a homogenous suspension. The suspension was transferred to a 100-mL Teflon-sealed autoclave and maintained at 160 °C for 4 h. The resultant TiO_2_/GQD nanocomposite was centrifuged, washed three times with distilled water and ethanol, and dried overnight in an 80 °C vacuum oven (Supplementary file).

### Characterization

X-ray powder diffraction* (XRD*) patterns were recorded on Bruker AXS D8-Advanced diffractometer in the 2θ range from 5° to 90° using Cu Kα (λ = 1.54056 Å) radiation. Fourier transform infrared (FT-IR) spectra were obtained on an ATI Unicam (Mattson936) Bench Top Spectrometer with pressed KBr pellets in the 4000–400/cm range. UV–Vis absorbance spectra were recorded using a Unico UV-2100 spectrophotometer at room temperature. The texture features of the obtained samples were estimated from N_2_ gas adsorption and desorption analysis measured at liquid nitrogen temperature (− 196 °C) using a Quantachrome Nova3200S apparatus. Before analysis, the samples were outgassed for 12 h at 250 °C, N_2_ atmosphere to remove moisture from the adsorbent surface. The specific surface area was calculated using the BET method, and the total pore volume was computed from the amount of adsorbed N_2_ at a P/P^o^ of 0.95. The pore size distribution curve was also calculated by the BJH method. HR-TEM image was performed on a JEOL-JEM 2100F instrument operated at 200 kV**.** Photoluminescence data (PL) were acquired at room temperature using Agilent Technologies (Cary Eclipse Fluorescence spectrophotometer).

### Photocatalytic performance

A 25 W UV lamp (type YB-25/T5, Genuine filter) was used to evaluate all samples for photocatalytic activity. The photoreactor employed was a 150-mL quartz glass vessel with an external jacket outside diameter of 4 cm, an internal jacket inside diameter of 3 cm, and a clear quartz tube of 25 cm in total length. To study the effect of adsorption and intermediation on the photocatalyst surface, the photocatalyst powder (0.05 g) was distributed in a glycerol solution (0.3 mol/L, 100 mL) using a mixture of solvents (25 mL of ethyl acetate and 15 mL of DI). To obtain glycerol’s perfect dispersion and adsorption–desorption equilibrium on the photocatalyst surface, the suspension was stirred continuously for 15 min at 500 rpm using a magnetic stirrer in the dark. After that, 30 mL of H_2_O_2_ was added to the solution before irradiation started. An aliquot of 5 mL was taken and filtered every half hour for analysis^30^. Quantitative analysis of the variation in glycerol concentration and chemical species in the samples was done using gas chromatography-mass spectroscopy (GC–MS). The Tauc-David-Mott equation can be used to determine the photocatalyst's band gap^29^.

1 $$ \alpha \, \left( {{\text{h}}\nu } \right)^{{{1}/{\text{n}}}} = {\text{ A}}\left( {{\text{ h}}\nu \, {-}{\text{ E}}_{{\text{g}}} } \right) $$

h is Planck’s constant, and A is the constant, respectively. The light frequency and the absorption coefficient, respectively, are denoted by λ and α. The exponential value n’s indicates the type of sample transition, being either 2 or 1/2 for permitted direct and indirect transitions. The following equations were used to calculate glycerol conversion efficiency (%XG LY) and product selectivity (%S), respectively^31^.

2 $$ \left( {glycerol \;conversion\left( \% \right)} \right) = \frac{ amount \;of \;glycerol \;converted }{{Total \;amount \;of \;glycerol\; in\; reactant }} *100 $$

3 $$ Selectivity \left( \% \right) = \frac{mol\; desired \;product}{{mol \;starting \;compound - mol \;starting \;compound \;left \;after \;reaction}} *100 $$

## Results and discussion

**Figure 1:**
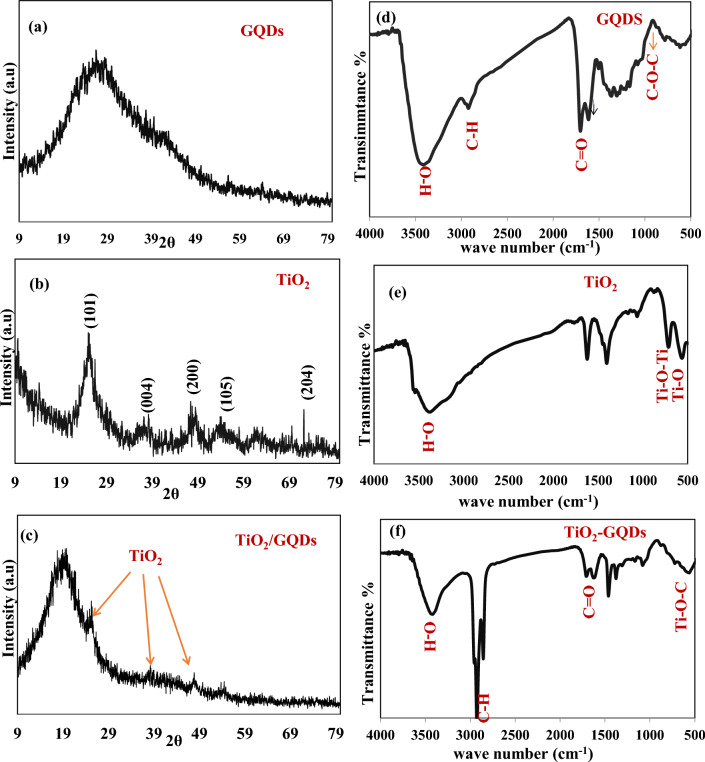
(**a**–**c**) XRD of GQDs, TiO_2_, and TiO_2_/GQDs nanocomposite, (**d–f**) FTIR of GQDs, TiO_2_, and TiO_2_/GQDs nanocomposite.

The crystallinity and phase structure of the prepared samples (TiO_2_, GQDs, TiO_2_/GQDs) are studied by XRD, as shown in Fig. 1a–c. The GQDs exhibited a broad peak with a maximum at ~ 29° that corresponds to the (002) of graphite lattice plane, according to (JCPDS No. 75-0444). The broad peak is owing to the small size of quantum dots^32^. For the TiO_2_ sample, the diffraction peaks appeared at 25.2°, 38°, 48.3°, 55° and 62.9° are attributed to (101), (004), (200), (105), and (204) tetragonal crystal plane of anatase TiO_2_ according to (JCPDS No. 04-0477)^29^. Furthermore, XRD analysis of the TiO_2_/GQDs sample has specific diffraction peaks at 19°, 26°, 39°, and 48°, confirming the presence of both TiO_2_ and GQDs. The shift in the diffraction peak of GQDs from 29° to 19° and that of TiO_2_ peaks from 25.2°, 38°, 48.3°, and 55° to 26°, 39°, and 48° indicate that TiO_2_ NPs and GQDs were successfully mixed. Moreover, the small intensity of TiO_2_ peaks may be attributed to incorporating TiO_2_ nanoparticles between GQD sheets.

FTIR was used to examine the chemical groups of GQDs and how they change when TiO_2_ NP is added to create TiO_2_/GQDs nanocomposite. For the GQDs sample, O–H stretching vibrational absorptions are responsible for the broad vibrations at 3416/cm (see Fig. 1d). The vibrational peak at 2924.3/cm represents the symmetric and asymmetric bond of C–H. Peaks at 617/cm connected to the (C–O–C) ester group, 1366/cm related to the (COO) carboxylic group, and 1700/cm related to the (C=O) carbonyl group all match with GQDs^33,34^. The FTIR spectrum of TiO_2_, Fig. 1e, showed broad absorption bands at 562/cm and 525/cm due to the vibrations of Ti–O–Ti and Ti–O-stretching, respectively. The GQDs peaks are still visible in the TiO_2_/GQDs sample, but with a change: the –OH group has changed the broad absorption band at 3416/cm into a sharp peak at 3418/cm compared to GQDs. The displacement of the band from 1700 to 1655/cm indicated that the complexation process involved the C=O stretching vibration of the COOH functional group. The interaction between GQDs and TiO_2_ NP led to the formation of the Ti–O–C bond at 542/cm, Fig. 1f. These results show TiO_2_ NP and GQDs have a strong reciprocal relationship^35^.

**Figure 2:**
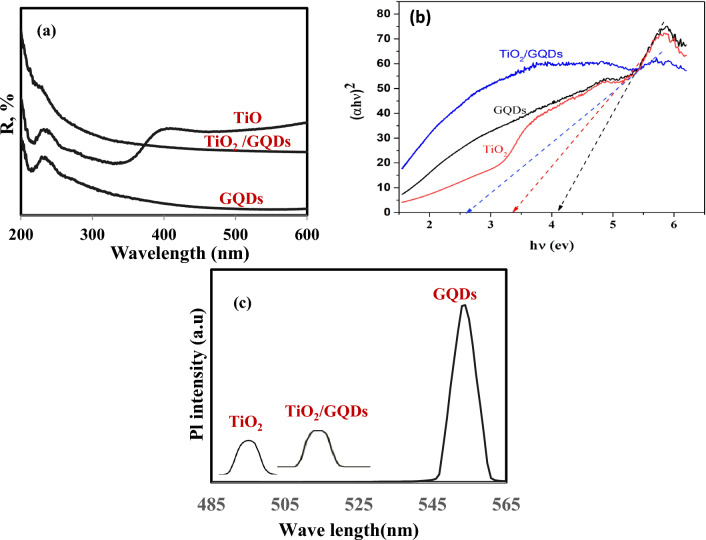
(**a**) UV–Vis spectra of GQDs, TiO_2_, nanocomposite TiO_2_/GQDs, (**b**) Tauc plots of the prepared photocatalyst, (**c**) PL spectra of GQDs, TiO_2_, nanocomposite TiO_2_/GQDs.

The samples of the UV–Vis absorption spectra of GQDs, TiO_2_, and TiO_2_/GQDs are presented in Fig. 2a. The UV–Vis absorption spectrum of the GQD sample shows a peak in the UV region at ~ 236 nm, which is assigned to the (π–π*) transition of the C=C aromatic bond of the sp^2^ hybridization domain^36^. While the TiO_2_ sample has two absorption spectra, one in the UV region (~ 240 nm) and another in the visible region (~ 400 nm), this unique behavior for nano-TiO_2_ and corresponds to the reported results^37^. In the meantime, the TiO_2_/GQD sample shows higher photo-absorption activity at a lower wavelength (at UV region), which decreases in the higher wavelength (of visible region > 400 nm)^38^. Moreover, the band gap of all samples is calculated from the Tauc/David-Mott formula^29^, as shown in the experimental section. Furthermore, each sample’s band gap is determined by the Tauc/David formula, which studies the relationship between (αhѵ)^2^ and hѵ. Figure 2b illustrates how to determine the gap energy by extrapolating the linear portion of (h)2 along the energy axis. TiO_2_/GQD sample has a lower band gap energy (2.6 eV), which demonstrates the compound’s semiconductor nature and high photoactivity^39^, compared to TiO_2_ NPs (3.2 eV) and GQDs (4.28 eV). This is most likely caused by the bonds that form when GQDs interact with TiO_2_ and/or the incorporation of TiO_2_ between GQD sheets, as confirmed by XRD analysis. This means that GQDs enhance photo-absorption due to Ti^3+^ formation, which generates local energy between the conduction band and the valence band of TiO_2_, leading to a decrease in band gap^40^.

The photoluminescence of TiO_2_, GQDs, and TiO_2_/GQDs was further studied to demonstrate the critical role of GQDs in allowing the transfer of photogenerated electrons to enhance the photocatalytic activity of TiO_2_/GQDs nanocomposites. As seen in Fig. 2c, TiO_2_ NPs displayed a characteristic emission peak at 500 nm from the material's excitonic band edge emission. The GQDs give off a peak emission at 550 nm. When GQDs were added to TiO_2_ NPs, the essential excited peak in the TiO_2_/GQDs was redshifted in comparison to pure TiO_2_, which can be attributed to the strong interaction between the GQDs and TiO_2_ and the formation of the Ti–O–C bond. This decrease in pl emission shows that the TiO_2_/GQDs emission decays are much faster than those of pure TiO_2_, demonstrating that the addition of GQDs provides a quick electron transfer channel for improved charge separation that enhances photocatalytic activity, as well as the well-known quantum confinement effect by shifting the conduction and valence band edges in opposite directions^41^ will improve the photocatalytic activity as well.

**Figure 3:**
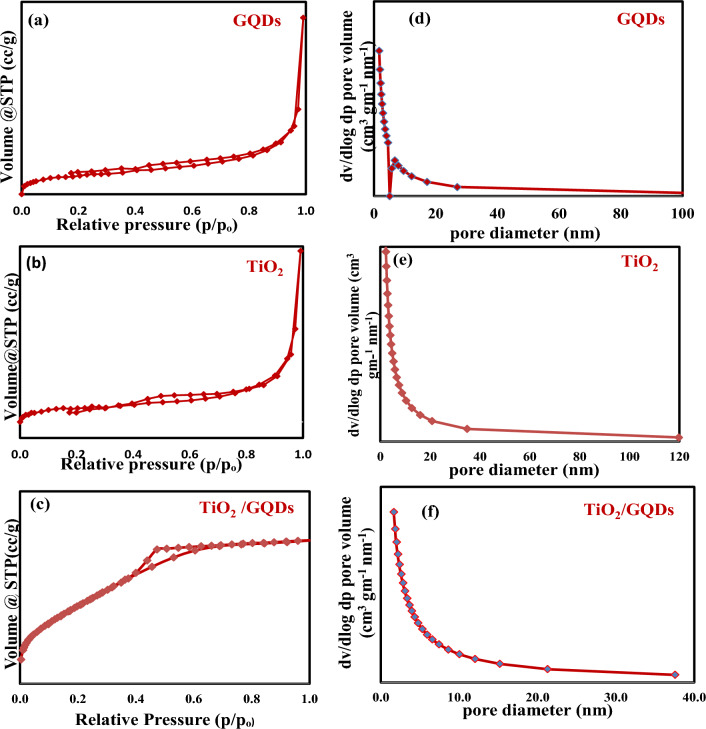
(**a**–**c**) The nitrogenadsorption–desorption of GQDs, TiO_2_, nanocompositeTiO_2_/GQDs, (**d**–**f**) pore diameter of GQDs, TiO_2_, nanocompositeTiO_2_/GQDs.

The predicted nitrogen adsorption–desorption isotherms and the BJH pore size distribution were plotted in Fig. 3a–c. TiO_2_ nanoparticles and GQDs exhibit a type II isotherm. In contrast, the nanocomposite sample exhibits a type IV adsorption isotherm with an H2 hysteresis loop, demonstrating the mesoporous structure of these samples and the presence of interconnected pores^42^. The samples’ pore sizes, which fall between 3 and 6 nm, further support their mesoporous nature. The respective total pore volumes of GQDs and TiO_2_ are 0.078 cc/g and 0.061 cc/g. The TiO_2_, GQDs, and TiO_2_/GQDs samples have specific surface areas of 33 m^2^/g, 21.7 m^2^/g, and 229 m^2^/g, respectively. The unexpectedly high BET surface area of TiO_2_/GQDs nanocomposite may be attributed to the formation of TiO–C chemical bonding between TiO_2_ and GQDs, which causes an increase in electronegativity in both TiO_2_ and GQDs, boosting N2 polarization and adsorption^43^. Increasing the surface area and pore volume of TiO_2_/GQDs after GQD loading will lead to more target molecules that can absorb and migrate to the photocatalyst surface, potentially boosting the photocatalytic effectiveness.

**Figure 4:**
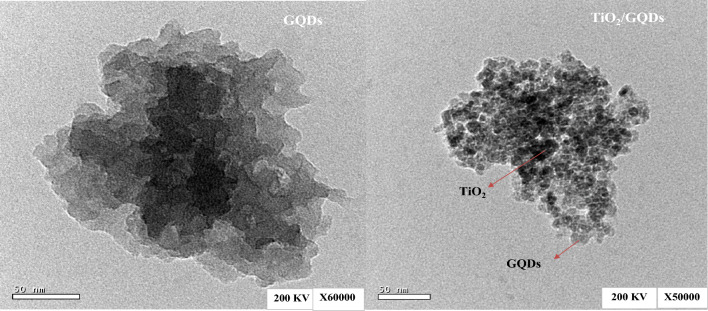
TEM images of GQDs, TiO_2_/GQDs.

TEM was used to examine the morphology of the GQDs, TiO_2_/GQDs in Fig. 4. GQDs have homogeneous nanosheets that are monodispersed. These are small chunks or fragments of graphene with lateral dimensions of less than 100 nm. The distinct contrasts between TiO_2_ and carbon atoms in GQDs confirm the formation of TiO_2_ /GQD.

### Photo-catalytic glycerol conversion

#### Effect of catalyst type and oxidant

Photocatalytic activities of the prepared samples; TiO_2_ NPs, GQDs, and TiO_2_/GQD nanocomposite were tested for glycerol conversion under UV irradiation at different reaction conditions; presence of oxidant, catalyst amount, reaction time, and solvent type. Firstly, the blank test was conducted under dark conditions for 30% glycerol aqueous solution in the presence and absence of catalysts, which showed no conversion. Another blank sample was tested in the presence of H_2_O_2_ as an oxidant in the dark, and no conversion was observed. Under UV irradiation in the absence of H_2_O_2_ and the presence of a TiO_2_ catalyst, only 0.5% of glycerol conversion was observed. Moreover, in the presence of H_2_O_2_ and the absence of a catalyst, only 1.05% glycerol conversion was observed.

**Figure 5:**
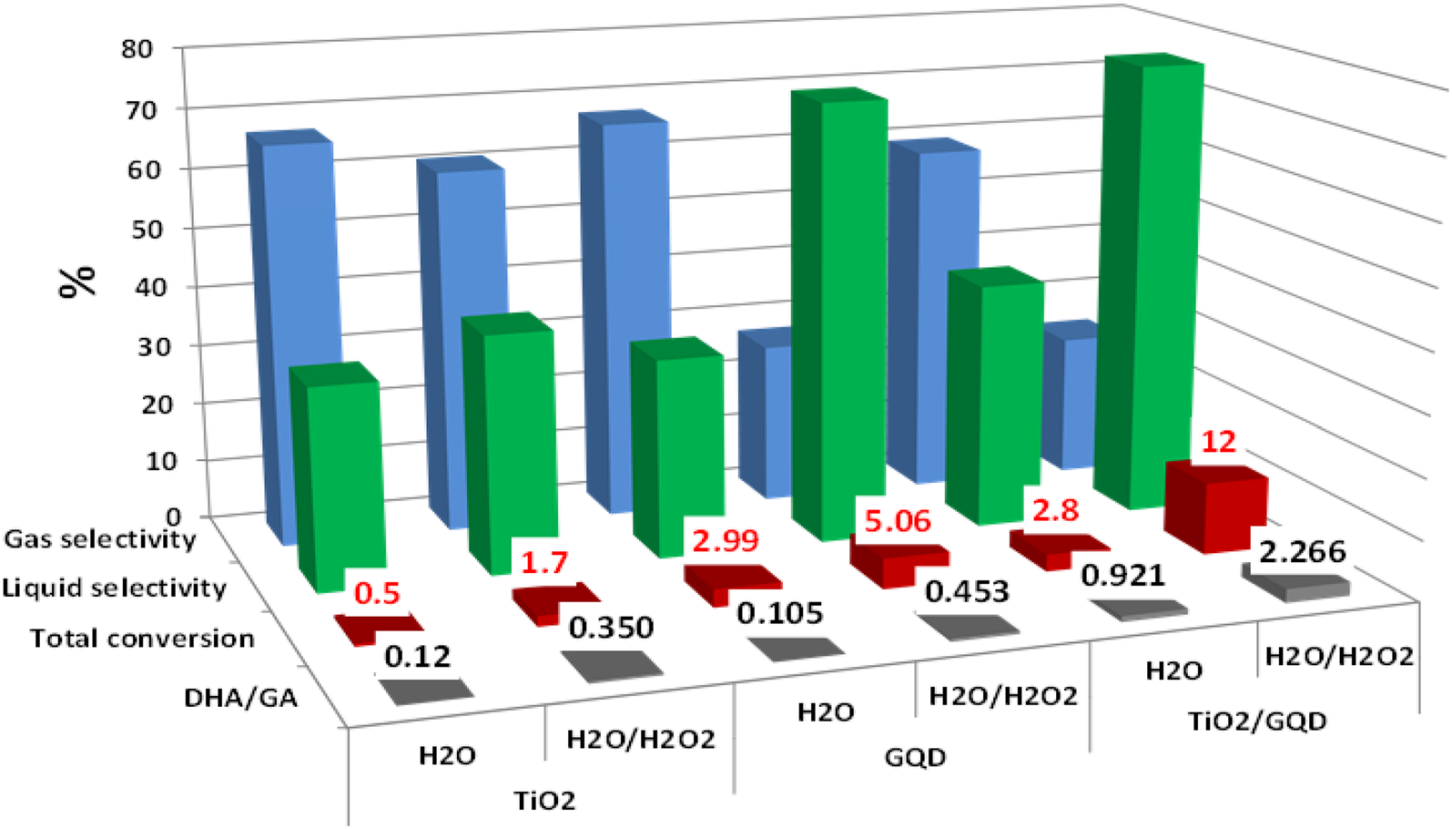
Effect of catalyst type (0.05 g) and presence of oxidant on photocatalytic glycerol conversion under 1h UV irradiation.

Figure 5 shows the effect of different photocatalysts (0.05 g) on glycerol conversion, liquid selectivity, gas selectivity, and DHA/GA ratio in the presence or absence of H_2_O_2_ under 1 h UV irradiation. According to GC–MS analysis, the liquid products consist of acetaldehyde, formaldehyde, acetone, glyceraldehyde (GAD), glycolic acid (GCD), propanediol and dihydroxyacetone (DHA). According to the literatures^44,45^, all those are value-added chemicals that can be used as feedstock in different industrial applications. However, the gas products are mainly H_2_ and CO_2_. This study focused on the total liquid and gas selectivity and the DHA/GA ratio as a common metric in photocatalytic glycerol oxidation, where it is important to improve the DHA selectivity as the most desirable product^46^. For the TiO_2_ NPs sample, only 0.5% conversion was observed in the absence of H_2_O_2_ and increased to 1.7% when the oxidant was added. In both cases, TiO_2_ NPs are more selective for gas products with a very low DHA/GA ratio within the liquid product. The inactivity of TiO_2_ can be attributed to the rapid recombination of photogenerated e^−^ h^+^ pairs. The GQDs sample shows slightly higher glycerol conversion either in the presence or absence of an oxidant, reaching 5.06% glycerol conversion in the presence of an oxidant. In the absence of an oxidant, the GQDs are selective to gas products, and in the presence of an oxidant, the liquid selectivity reaches 73% with a DHA/GA ratio of ~ 0.45. These data are is also not satisfactory, and it can be attributed to the slightly high energy gap (4.28 eV) of parent GQDs as the high band gap of GQDs makes an obstacle for electron transfer in between GQDs sheets and increases the activation energy of reaction to occur, which makes the conversion of glycerol to selective product insatisfactory^47^.

**Figure 6:**
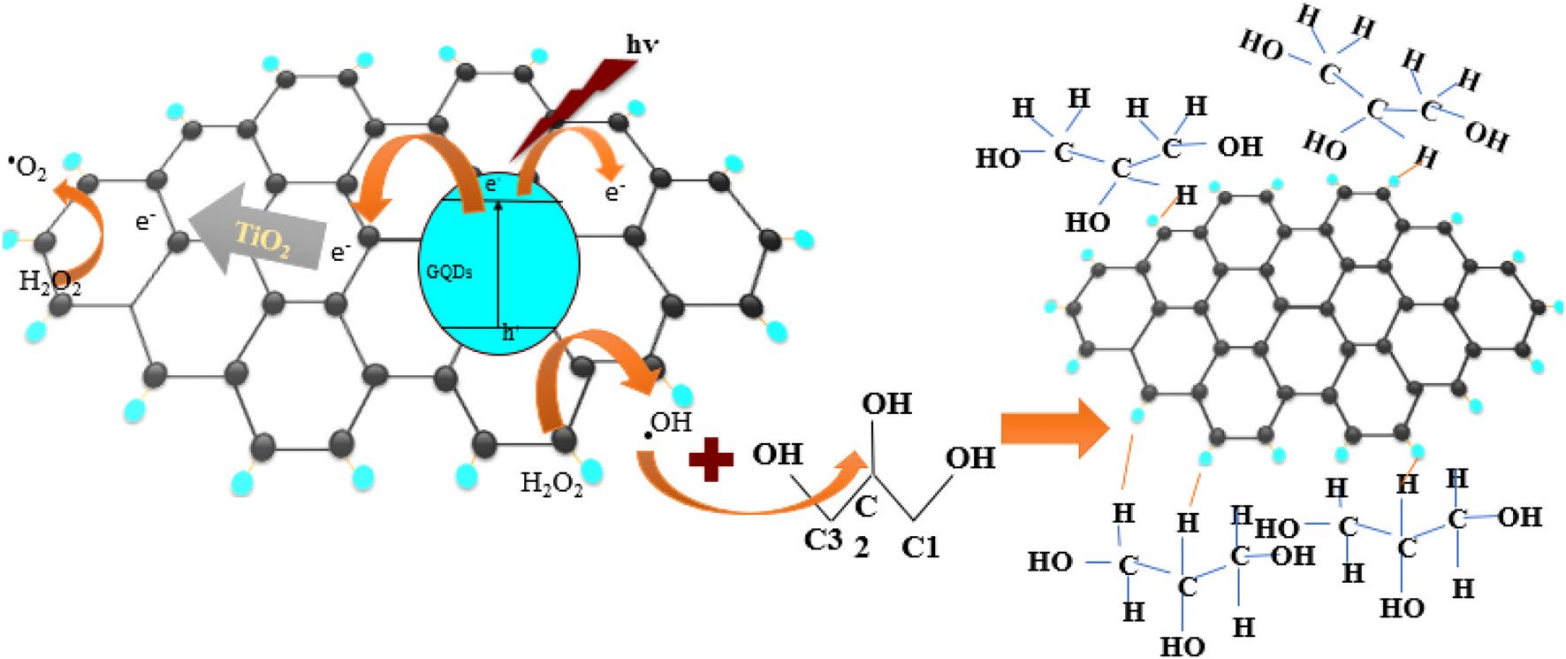
The pathway for the migration of electrons from the catalyst's surface during photocatalytic glycerol oxidation to the liquid product.

However, combining two materials to form a TiO_2_/GQD nanocomposite increases the photocatalytic efficiency either in the presence or the absence of H_2_O_2_. The photocatalytic glycerol conversion in the presence of H_2_O_2_ and TiO_2_/GQD shows the highest glycerol conversion (12%) with low selectivity toward gas products (23%), high selectivity liquid products (76%) and DHA/GA ratio ~ 2.26. The glycerol conversion over TiO_2_/GQD catalyst was 7 times more than TiO_2_ NPs and 2.3 times more than GQDs under the same reaction conditions. This can be attributed to the combination of geometrical and optical characteristics in the new material, which poses a high surface area, good ability for light absorption, and low band gap energy, as shown in the “Characterization” section. The high surface area of TiO_2_/GQD nanocomposite leads to a high glycerol adsorption rate on the catalyst surface and increases light absorption. This intense interaction between GQD and TiO_2_ leads to the formation of closely contacting structures (CCS). The CCS can enhance the role of synergistic effect between TiO_2_ and GQD in photocatalytic oxidation of glycerol, as shown in Fig. 6. Moreover, the GQD surface is rich with different function groups (as confirmed by FTIR) such as hydroxyl group (-OH), carboxyl group (COOH), and carbonyl group (C=O) located either on its surface and/or on its edges. So, the glycerol molecules can attract easily and with high concentration to the catalyst GQD surface, forming a hydrogen bonding network with different sites. This hydrogen bond strongly tightens the glycerol on the catalyst surface either via a single hydrogen bond or chelated hydrogen bonding between two hydroxyl groups of glycerol with the surface-active sites on GQDs^48^.

Furthermore, the good ability of light absorption of TiO_2_ activates the catalyst to form charge carriers. The high carrier mobility (due to CCS) and unique zero-dimensional effect of GQDs can facilitate electron transfer (TiO_2_/GQDs + hυ → hvb^+^  + ecb^−^) and inhibit e^−^–h^+^ recombination^49^. So, the photo-generated electrons (e^−^), due to TiO_2_ excitation, can flow to the GQDs surface via the TiOC bond. The e^−^ can react with a solvent in the solution, forming O^•2−^ and/or ^•^OH (Eqs. 4–6). Finally, the radicals attack the adsorbed glycerol molecules, leading to glycerol dehydrogenation, forming different products, as shown in Fig. 6.

On the other hand, the positive effect of oxidant addition can be explained as follows: glycerol oxidation can proceed via the interaction with various oxidizing species, photogenerated holes (h^+^), hydroxyl radicals (HO^-^), superoxide ion radicals (O^−2^) and hydrogen peroxide (H_2_O_2_)^31^. Under UV irradiation, in the presence of catalysts dispersed in pure H_2_O without oxidant addition, photogenerated holes (h^+^) are produced^50^. That can facilitate the complete oxidation of glycerol to H_2_ and CO_2_ gases. The GQDs on the TiO_2_ surface can trap the photoinduced e^−^ from TiO_2_ CB, efficiently separating the e^−^ and h^+^ and enhancing the photocatalytic H_2_ evolution. Moreover, the π-conjugated GQDs are also excited by absorbing light photons, which act as a photosensitizer to sensitize TiO_2_ and then transfer photo-excited electrons from the GQDs to the conduction band of TiO_2_ to produce H_2_, thereby improving the gas selectivity^51^.

On the other hand, when the oxidant was added, H_2_O_2_ absorbed the UV light and split into ^•^OH radicals, which also can oxidize glycerol. So, in this case, the conversion of glycerol and generation of products can proceed via either h^+^-mediated (from catalyst) or radical-mediated (from oxidant) routes. The ^•^OH radicals can be generated either by the reduction of H_2_O_2_ with photogenerated electrons (e^−^) in the conduction band (H_2_O_2_‏ + e^−^ → OH^−^ + ‏ ^•^OH) or photogenerated holes (h^+^) in the valance band (H_2_O_2_ ‏ + h^+^  → 2^•^OH)^51^. The H_2_O_2_ can further oxidize to form O^•2–^by holes in the presence of OH^−^ and/or ^•^OH (Eqs. 4–6). Also, the ^•^OH can be produced from H_2_O by the effect of photogenerated holes (hvb^+^  + H_2_O → ^•^OH + H^+)^. The high increase in glycerol conversion in the presence of both H_2_O_2_ and TiO_2_/GQDs photocatalysts indicates that the radical-mediated route was the dominant mechanism, where the generated ^•^OH radicals had more oxidizing power than the photogenerated holes^52^. The liquid products are formed due to the photocatalytic oxidation of glycerol due to hydroxyl radical (^•^OH) photogeneration, as shown in Fig. 6. Thus, the excess ^•^OH radical in the reaction solution is responsible for the high selectivity for liquid product^40^. It is also worth noting that the formation of chelation hydrogen bonds in this system may facilitate the dehydrogenation of glycerol via the C2 position, which increases DHA selectivity over GAD (Fig. 6).

4 $$ {\text{H}}_{{\text{2}}} {\text{O}}_{2}  + 2{\text{OH}}^{ - }  + {\text{ h}}^{ + }  \to {\text{ O}}_{2}^{{ \cdot  - }}  + 2{\text{H}}_{{\text{2}}} {\text{O}}  $$

5 $$  {\text{H}}_{{\text{2}}} {\text{O}}_{{\text{2}}} {\text{ }} + {\text{ OH}}^{ - } {\text{ }} + ^{ \cdot} {\text{OH}} \to {\text{ O}}_{2}^{{ \cdot  - }}  + 2{\text{H}}_{2} {\text{O}}   $$

6 $$   {\text{H}}_{{\text{2}}} {\text{O}}_{{\text{2}}} {\text{ }} + {\text{O}}_{2}^{{ \cdot  - }}  \to ~~{\text{OH}}^{ - } {\text{ }} + ^{ \cdot} {\text{OH}} + {\text{ O}}_{{\text{2}}}  $$

#### The effect of catalyst amount and time of irradiation

**Figure 7:**
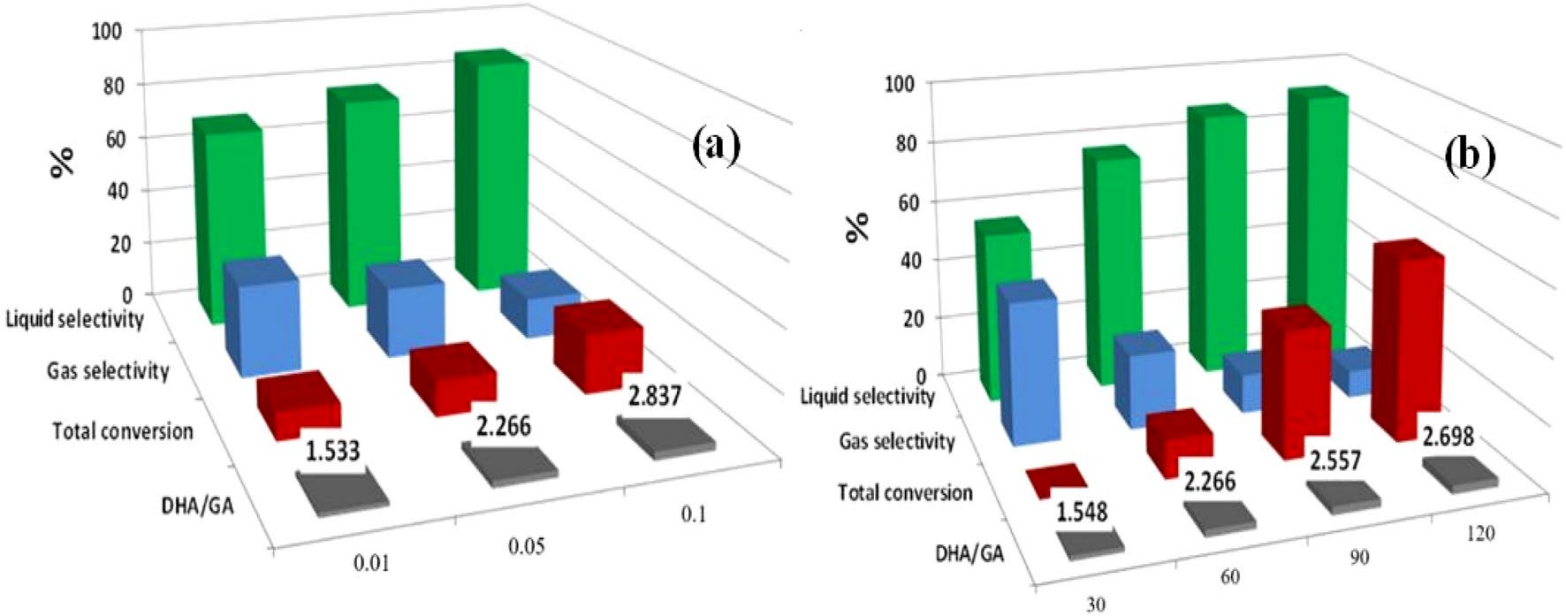
Effect of reaction condition on glycerol conversion and product selectivity.

The effect of TiO_2_/GQD catalyst amount was also studied in the presence of H_2_O_2_ oxidant for 1 h irradiation (Fig. 7a). The data indicate that the increase in photocatalyst amount slightly affects the glycerol conversion to a liquid product of high selectivity. The glycerol conversion reaches 20% with 86% liquid selectivity containing ~ 2.8 DHA/GA when using 0.1 g TiO_2_/GQD. This can be due to the combination of TiO_2_ NPs with the ultrafine quantum dots of graphene sheets providing a larger active surface, which increases the contact area and accelerates the rate of photo-generated electron transfer.

Moreover, the effect of irradiation time has a significant effect on glycerol conversion during the photocatalytic oxidation process. It was studied using 0.05 g of TiO_2_/GQDs in the presence of H_2_O_2_ oxidants (Fig. 7b). It was found that the conversion of glycerol began after 1 h irradiation time, suggesting that a small number of photogenerated holes were generated during this time and cannot lead to effective glycerol conversion. Moreover, after that, the glycerol conversion highly increased along the irradiation time until it reached 57% after 2 h with 91% selectivity for liquid products. That can be attributed to the excellent electron transport properties and the quantum confinement effect of photocatalysts.

#### The effect of solvent type

**Figure 8:**
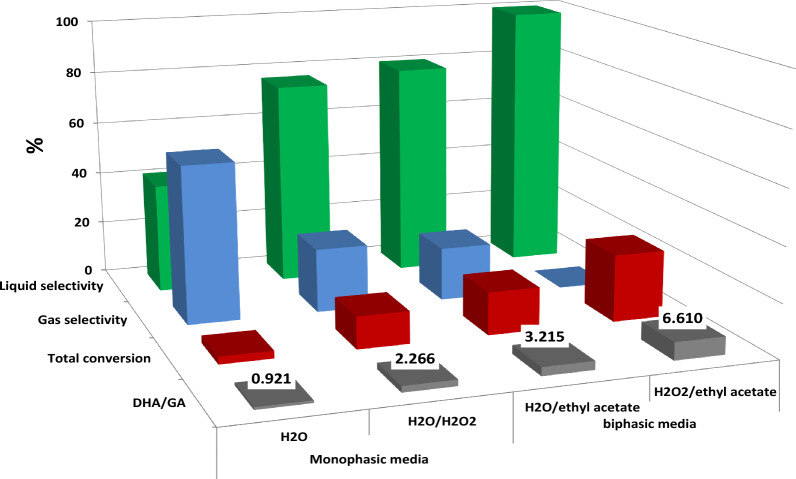
Effect of biphasic photoreactor.

Another critical factor that significantly affects glycerol conversion is using various solvents. Herein, the use of biphasic media for photocatalytic oxidation reaction was studied using a mixture of aqueous phase (H_2_O or H_2_O/H_2_O_2_) and organic phase (ethyl acetate) with a ratio of 1:2.5 (Fig. 8). Comparing the glycerol conversion, liquid and gas selectivity under identical conditions using water monophasic media and water/ethyl acetate biphasic one indicates that, the glycerol conversion in biphasic media increase to ~ 15.9% with ~ 80% liquid selectivity containing DHA/GA ratio of ~ 3.2. The DHA selectivity is higher in biphasic media, but this still suffered from a slightly low DHA selectivity.

After adding two phases, it was noticed that small droplets of water were formed and dispersed in the ethyl acetate phase immediately, and the photocatalyst had excellent dispersion in the reaction system. This can be attributed to the hydrophilic nature of the TiO_2_/GQDs, so it remains within the dispersed phase (H_2_O) because it strongly prefers water. The data in (Fig. 8) indicate that the glycerol conversion highly increases in biphasic media than in monophasic ones, which can be attributed to the solubility of glycerol in water is much higher than that in ethyl acetate (1 part of glycerol dissolves in 11 parts of ethyl acetate^53^). The biphasic reactor comprises numerous small micro-reactors (aqueous droplets) containing glycerol and a photocatalyst, where the photocatalytic glycerol oxidation occurs almost in those micro-reactors (droplet phase). On the other hand, ethyl acetate has negligible light absorbance, so a higher light intensity was affected in water droplets and more efficient light delivery to the photocatalyst than in the monophasic reactor^28^. This means biphasic media improves light penetration into the microreactor, increasing the photocatalytic glycerol oxidation reaction rate.

Moreover, the effect of adding an oxidant (H_2_O_2_) to the biphasic media was also studied herein. In this experiment, 15 mL glycerol, 5 ml deionized water, 25 mL ethylacetate, 5 mL H_2_O_2_, and 0.05gm TiO_2_/GQDs were added. The glycerol conversion data are in Fig. 8 indicates that the use of H_2_O_2_ as an oxidant in the biphasic photoreactor gives the highest glycerol conversion (25%) with ~ 99.8% liquid conversion containing a high DHA/GA ratio (~ 6.6). Those data seem better than that of the monophasic reactor using H_2_O_2_ as an oxidant and more than that of a biphasic reactor without an oxidant, which may be related to the dense photon absorption in the microreactors facilitating the high ^•^OH radical generation that induces the glycerol conversion to liquid products.

Table 1 compares the performance of different TiO_2_ photo-catalysts in glycerol conversion and their selectivity toward liquid and gas products reported in the literature. Most catalysts consist of TiO_2_ as support doped with noble or non-noble metals. In contrast, the current catalysts consist of TiO_2_ supported on cheap/available support as GQD prepared from glucose powder. In addition, the reported catalysts show glycerol conversion did not exceed in aqueous solution after 5 h. However, TiO_2_/GQD shows the same conversion after 90 min with liquid product selectivity (S) ~ 87% having 2.5 of DHA/GA. Furthermore, ~ 57% glycerol conversion was observed after 120 min of the same condition. Moreover, using biphasic media (H_2_O/ethyl acetate) the DHA/GA ratio reaches ~ 6.6. The aim is to explore the possibility of preparing active photo-catalysts from cheap and available precursors for a pilot scale for photo-conversion of glycerol (biomass derivatives) to value-added products with ultrahigh selectivity.

**Table 1:** Comparison of photo-catalytic glycerol conversion with reported data.

Catalyst	Preparation method	Light source	solvent	Reaction condition	Glycerol conversion	Liquid product	Gas product	References
TiO_2_ anatase	Commercial	UV high-pressure mercury lamp	Water	Time: h Cat. Wt.: g oxidant: H_2_O_2_	71.20%	GAD, GCA & DHA	–	^31^
						S_DHA_ = 23.6%		
						S_GAD_ = 68.2%		
						S_GCA_ = 8.16%		
TiO_2_	Hydrothermal	Xe-arc lamp	Acetonitrile	Time: h Cat. Wt.: g Oxidant: –	96%	GAD, GCA & DHA	–	^54^
						S_DHA_ = 17.8%		
			Water		36%	S_DHA_ = 14.7%		
Pt-TiO_2_	Deposition	Hg lamp	Water	Time: h Cat. Wt.: g Oxidant: –	40.60%	GAD & DHA	–	^55^
						S_DHA_ = 11.4%		
5wt% WO_3_/TiO_2_	Hydrothermal	UV lamp	Water	Time: h Cat. Wt.: g Oxidant: –	45%	GAD, DHA, GCA, OA, FA & AA	C_co2_ < 5%	^56^
						S_GAD_ = 29%		
3 wt% Cu_2_O/TiO_2_	Ball Mills	Hg lamp	Water	Time: h Cat. Wt.: g Oxidant: –	33%	GAD & DHA	C _H2_ = 1.7	^55^
						S_DHA_ = 10.3%		
						S_GAD_ = 5.4%	Cco_2_ = 1.7	
Ag-AgBr/TiO_2_	Photo-deposition	Xe-arc lamp	Acetonitrile	Time: h Cat. Wt.: g Oxidant: –	65%	GAD & GCA	–	^57^
						S_GAD =_ 37%		
TiO_2_/GQD	Hydrothermal	Xe-arc lamp	Water/Ethyl acetate	Time: h Cat. Wt.: g Oxidant	68%	S_DHA_: S_GA_ ratio = 4.3	–	^58^
TiO_2_/GQD	Hydrothermal	UV lamp	Water	Time: h Cat. Wt.: g Oxidant: H_2_O_2_	42%	GAD, DHA, GCA, OA, FA & AA	H_2_ CO_2_	Current work
						S_DHA_ = 19% S_GA_ = 18%		
				Time: h Cat. Wt.: g Oxidant: –	10%	S_DHA_: SGA ratio = 3.2		
			Water/Ethyl acetate/H_2_O_2_	Time: h Cat. Wt.: g Oxidant	87%	S_DHA_: S_GA_ ratio = 6.6		

GAD, glyceraldehyde; DHA, dihydroxyacetone; GCD, glycolic acid; OA, oxalic acid; FA, formic acid; AA, acetic acid; S, liquid product Selectivity; C, gas product concentration.

In conclusion, this work presents a facile way to prepare cheap (noble metal-free) photo-catalysts for glycerol conversion in a monophasic or biphasic photoreactor to produce ultrapure dihydroxyacetone. The TiO_2_/GQDs composite is successfully formed with a relatively high surface area (253 m^2^/g) via the hydrothermal method. The UV–Vis analysis confirmed a lower band gap for TiO_2_/GQDs than TiO_2_ and GQDs, which can enhance photoabsorption properties. The most important achievement in this research is photocatalytic glycerol oxidation in biphasic media (aqueous/organic phases) using H_2_O_2_ as an oxidant, which gives liquid selectivity of ~ 99.8% and DHA/GA ratio ~ 6.6, regarded as high in comparison to earlier studies. This study confirms that the use of non-aqueous solvents has a positive effect on photocatalytic processes. Also, its role in glycerol conversion and control over the final product (ultrapure DHA) gives it a good advantage in industrial applications. In contrast, DHA may be obtained in pure form without the use of purification techniques and is employed in various processes, including self-tanning, food preparation, cosmetic product manufacturing, and the manufacturing of polymers.

### Supplementary Information

Supplementary Information.

## Data Availability

The datasets used and/or analyzed during the current study are available from the corresponding author (Sara Hassan) upon reasonable request.
