# Supplementary material for: Clarifying solvent effect during photocatalytic glycerol conversion on TiO2/GQD as selective photocatalyst
Source: Sci Rep. 2023 Dec 9;13:21820. doi: 10.1038/s41598-023-48781-3 (PMC10710419; doi:10.1038/s41598-023-48781-3)
Supplement: Supplementary file 1 — Supplementary Information. [file 41598_2023_48781_MOESM1_ESM.docx]

**Supplementary FiLe**

**Clarifying solvent effect during photocatalytic glycerol conversion on TiO_2_/GQD as selective photocatalyst**

**Sara Hassan^a,^* , Dalia R. Abd El-Hafiz^a^, E.S. Abdullah^a^, Mostafa M.H. Khalil^b^**

^a^ Egyptian Petroleum Research Institute EPRI, Nasr City, Cairo, Egypt

^b^ Chemistry Department, Faculty of Science, Ain Shams University, 11566, Abbassia, Cairo, Egypt

**Figure s1**

**
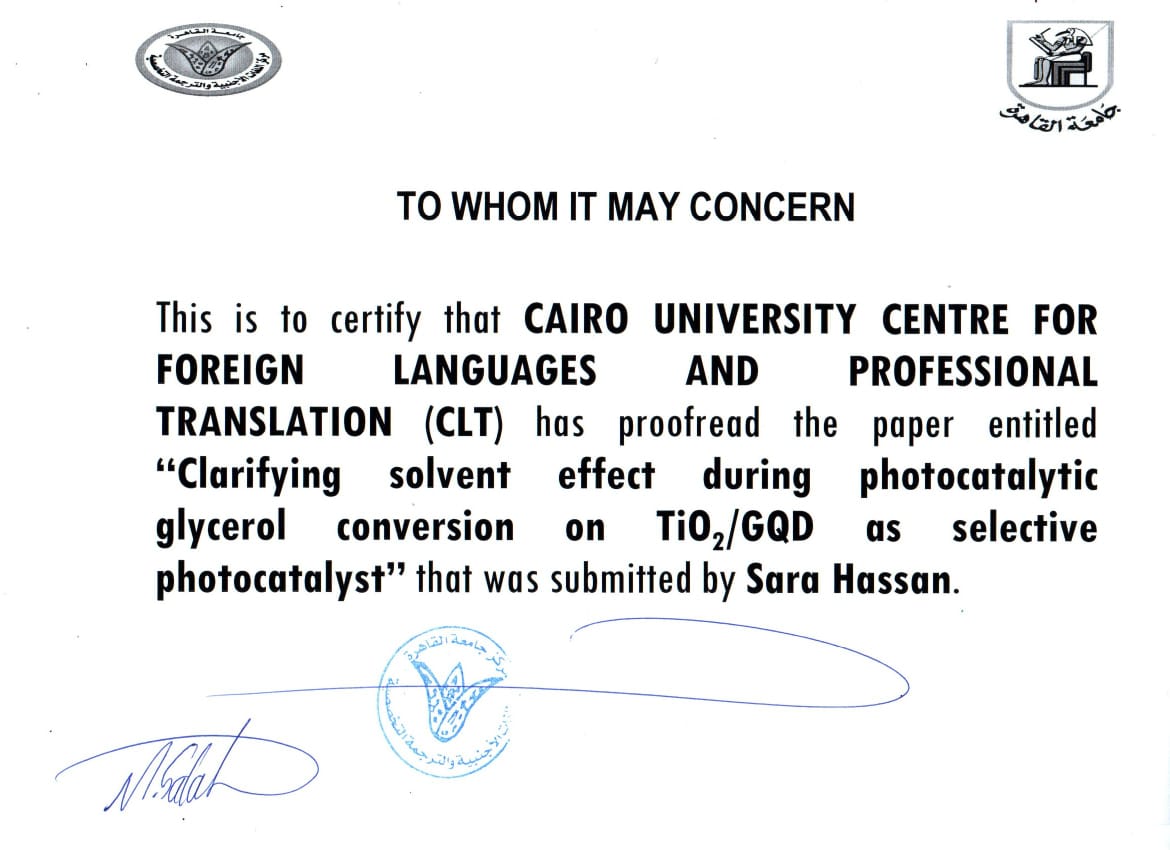
Equation S1**

**liquid selectivity % = carbon in liquid /glycerol conversion *100**

**Equation S2**

**gas selectivity % = 100- liquid selectivity %**
